# Musculoskeletal manifestations in a cohort of Behçet’s disease patients and their impact on health-related quality of life

**DOI:** 10.1007/s11739-025-03903-9

**Published:** 2025-03-07

**Authors:** Samar Tharwat, Nasim Jaber, Hamza Aljubaeh, Omar Abumunshar

**Affiliations:** 1 Rheumatology and Immunology Unit, Department of Internal Medicine, Faculty of Medicine, Mansoura University Hospital, Mansoura University, El Gomhouria St, Mansoura, 35511 Dakahlia Governorate Egypt; 2 Department of Internal Medicine, Faculty of Medicine, Horus University, New Damietta, Egypt; 3 Faculty of Medicine, Mansoura University, Mansoura, Egypt

**Keywords:** Musculoskeletal, Pain, Health-related quality of life, Behçet’s disease

## Abstract

Behçet’s disease (BD) is a multifaceted disorder of undetermined etiology. Distinct clinical manifestations exhibit varying prevalences, with mucocutaneous and ocular presentations being the most prevalent in the BD population. The aim of this study was to assess musculoskeletal (MSK) manifestations and their effect on health-related quality of life (HRQoL) of life in individuals with BD. We asked patients with BD to complete an online survey. The survey had many questions focused on demographic, clinical, and therapeutic data, as well as the Nordic musculoskeletal questionnaire and the short form-36 (SF-36). There was a total of 185 BD patients, mostly females (54.6%), with a mean age of 33.81 years. The most prevalent clinical manifestation was recurrent oral aphthosis (95.1%), followed by ocular involvement (72.4%). Most of the study patients (85.4%) reported MSK manifestations in the last 6 months. These manifestations included mainly the lower back (69%) and neck (67%), followed by the left and right knees (62% and 60%, respectively), while the least affected areas of the body were the right elbow (37%), and the right ankle and foot (7%). The age at disease onset (p = 0.007) showed a statistically significant difference between those with MSK manifestations and those without. Patients exhibiting MSK manifestations demonstrated statistically significant lower scores of all SF-36 domains compared to those without such manifestations. MSK manifestations are prevalent and adversely affect HRQoL among BD patients. Therefore, early identification and treatment are strongly recommended.

## Introduction

Behçet disease (BD) is a primary systemic vasculitis of indeterminate origin that impacts both large and small blood vessels within the venous and arterial systems [1]. It is more common along the historic trade route termed the “Silk Road,” which extends from the Mediterranean to the nations of the Far East [2]. Epidemiologic studies, which have been conducted in numerous countries worldwide, have reported highly variable prevalence estimates for BD. These estimates range from 0.1 per 100,000 in Hawaii [3] to 664 per 100,000 in Northern Jordan [4]. The cause of BD is uncertain; however, genetic factors, specifically HLA-B51 carriage, may play an important role. The environmental risk determinants are not well understood [5].

One of the distinguishing characteristics of BD is its heterogeneity, as the clinical manifestations and severity of the disease are not always consistent among patients [6]. BD is a multisystemic condition that can manifest by cutaneous lesions, ophthalmic involvement, gastrointestinal and central nervous system abnormalities, and other pathologies [7]. Musculoskeletal (MSK) involvement in BD is prevalent; however, the frequency of joint involvement differs significantly based on the geographic location [8] and study design, as well as evaluating medical subspecialties and the definition of involvement (arthritis vs. arthropathy). The prevalence is typically around 50%, with a range of 39 to 70% [9].

The Nordic MSK Questionnaire (NMQ-E) is a standardized tool originally designed to assess musculoskeletal (MSK) symptoms in occupational health settings in Nordic countries [10] but has since been widely adapted for use in various nations, including Arabic-speaking populations and clinical populations, including those with rheumatic diseases. Its structured format enables a systematic evaluation of MSK symptoms across different anatomical regions, making it a valuable tool for identifying symptom distribution and burden in chronic inflammatory conditions [11, 12]. In BD, MSK symptoms—such as arthritis, enthesitis, and myalgia—are frequently reported and can be a major contributor to disease burden [13]. Despite its initial use in occupational health research, the NMQ has been used in rheumatologic settings, demonstrating its effectiveness in capturing MSK involvement in conditions. Its comprehensive and systematic approach makes it an appropriate choice for assessing the extent, location [14], and impact of MSK manifestations in BD, where joint-related symptoms remain understudied.

Health-related quality of life (HRQoL) has emerged as a significant outcome variable in patients with chronic conditions [15]. By definition, HRQoL refers to a person’s capacity to meet their own basic needs, be satisfied with their lives, maintain a sufficient degree of social, interpersonal, and professional connections, and ultimately attain emotional and physical well-being [16]. The Short Form-36 (SF-36), the most widely used generic HR-QOL measure, was administered [17].

The assessment of HRQoL in patients with BD has primarily served as a surrogate for disease outcomes for many years [18]. Nonetheless, a limited number of studies have investigated the MSK manifestations in patients with BD and their impact on HRQoL. So, the aim of this study was to assess MSK manifestations and their effect on HRQoL of life in individuals with BD.

## Patients and methods

### Study design and setting

This cross-sectional observational analytic study was conducted on 185 people with BD. The study relied on surveys, requiring participants to complete a self-administered online questionnaire developed using Google Forms. The study included all adult patients with BD, diagnosed according to the International Study Group for BD [19], with confirmation from an expert rheumatologist.

To ensure the accuracy of the clinical data provided in the survey, participants were required to report on their BD-related manifestations and treatments as documented in their medical records. Where necessary, participants were encouraged to verify their responses with their treating physicians before submission. Individuals diagnosed with malignancies or any chronic rheumatic, MSK, or neurological diseases previous to the onset of BD were excluded from participation in the study from the outset. The questionnaire was randomly disseminated to all potential participants through social media platforms, including Facebook and WhatsApp, from August to November 2024. Subsequently, participants were sent to a webpage that outlined the study's objective and furnished instructions for completing the questionnaire. Participants were guaranteed anonymity and the confidentiality of their data. All individuals who consented to participate in the study were instructed to access Google Form. It was acknowledged that answering all questions and thereafter submitting them constituted consent to participate in the study.

### Sample size calculation

The sample size calculation was performed using G*Power, with the primary outcome being the prevalence of MSK manifestations among BD patients, estimated at 51.2% [8]. The effect size was set at − 0.1, with an alpha error of 0.05 and a study power of 0.80. Based on these parameters, the required sample size was determined to be 159 participants. To account for a potential 10% dropout rate, the final sample size was adjusted to 175 participants.

### Ethical consideration

This study adhered to the principles of the Helsinki Declaration, and the study protocol was approved by the Institutional Research Board of the Faculty of Medicine at Mansoura University (approval registration number: R.24.11.2881).

### Sociodemographic characteristics

All participants were asked to provide responses about sociodemographic data, including age, gender, marital status, level of education, and employment status. Furthermore, any habits of medical significance, such as smoking and alcohol consumption, were also reported.

### Clinical data

Clinical data on BD was also acquired from the participants. They were interrogated regarding the age at onset of BD, disease duration, and whether there was any diagnostic delay. Cumulative clinical manifestations of BD were also documented, including mucocutaneous, constitutional, gastrointestinal, ocular, central nervous system, and cardiac manifestations.

### BD therapeutic data

Therapeutic data was also acquired from the participants. They were questioned regarding the pharmacological agents employed to manage their BD, encompassing corticosteroids, colchicine, conventional disease-modifying antirheumatic drugs (cDMARDs), and biological DMARDs (bDMARDs).

### The Nordic MSK questionnaire

The principal objective of the NMQ-E was to evaluate the occurrence and distribution of MSK symptoms comprehensively across various anatomical areas. The participant was requested to identify the approximate location of the body areas causing discomfort within the last 6 months [20]. We also documented the duration of MSK discomfort..

The Arabic version of this questionnaire was utilized in this study. It has undergone cultural adaptation and validation, ensuring its reliability and applicability for Arabic-speaking populations [21].

### Short form 36 (SF-36)

SF-36 is a self-administered questionnaire that assesses HRQoL across eight domains of subjective health. The 36 individual items include eight subscales, with lower scores indicating poorer health (range 0–100). Norm-based scores were utilized. There are eight subscales: physical functioning (PF), role limitation due to physical functioning (RP), bodily pain (BP), general health (GH), vitality (VT), social functioning (SF), role limitation due to emotional problems (RE), and mental health (MH). The Arabic version of SF-36 was used as it has been validated for use in Arabic-speaking populations [22].

### Statistical analysis

The analysis of the acquired data was conducted using the Statistical Package for Social Sciences (SPSS) version 22. Quantitative data was presented using mean and standard deviations (SD) for parametric variables, and median (min–max) for nonparametric variables. In presenting qualitative data, we utilized percentages and numerical values. We conducted the Shapiro–Wilk test to determine the normality of the variable’s distribution.. The independent samples t-test was employed to ascertain if a statistically significant difference existed between two groups under normally distributed data; conversely, the Mann–Whitney test was applied when the variables were non-parametric. We employed either the Chi-square test or the Fisher exact test for comparisons including qualitative variables, as appropriate. A p value below 0.05 was deemed statistically significant.

## Results

**Fig. 1:**
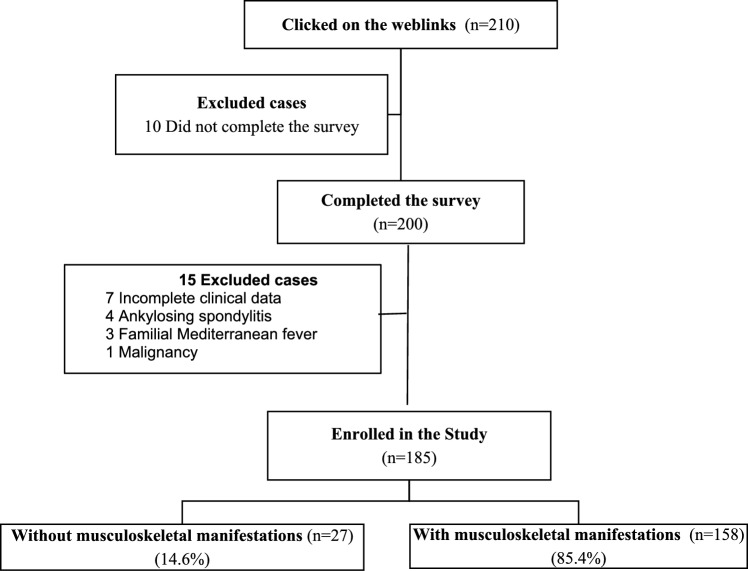
Flowchart of the study

Throughout the study, we contacted 300 individuals diagnosed with BD, inviting them to participate in the online survey. Of these, 210 individuals clicked on weblinks directing them to the survey; however, only 200 completed it. Fifteen participants were excluded for multiple reasons: seven lacked clinical data, four were diagnosed with ankylosing spondylitis, three had concomitant familial Mediterranean fever, and one had malignancy prior to the onset of BD. Participants were categorized into two groups based on the occurrence of MSK symptoms in the preceding 6 months. The flowchart of the study is illustrated in Fig. 1.

The study included a total of 185 BD patients. The mean age (SD) was 33.81 (9.32) years; most of them were females (54.6%) and married (61.6%). Graduates made up about one-third (36.8%), while employed individuals made up about half (52.4%). Nearly one fifth were smokers (21.1%). There was no statistically significant difference in employment status and life habits between individuals with MSK manifestations and those without. However, individuals with MSK manifestations had a statistically significant higher age (p = 0.034). Additionally, MSK manifestations were more prevalent in females (p < 0.001), as shown in Table 1.

**Table 1:** Baseline demographic characteristics of the study BD patients (n = 185)

Variable n (%), median (min–max), mean ± SD	Total BD patients (n = 185)	Musculoskeletal manifestations		p
		Without (n = 27) 14.6%	With (n = 158) 85.4%	
Age (years)	33.81 ± 9.32	30.23 ± 8.55	34.42 ± 9.34	**0.034**
Gender				
Male	84 (45.4)	24 (88.9)	77 (48.7)	** < 0.001**
Female	101 (54.6)	3 (11.1)	81 (51.3)	
Country				
Marital status				
Single	64 (34.6)	15 (55.6)	49 (31.0)	0.081
Married	114 (61.6	12 (44.4)	102 (64.6)	
Widow	5 (2.7)	–	5 (3.2)	
Divorced	2 (1.1)	–	2 (1.3)	
Living alone	8 (4.3)	–	8 (5.1)	0.606
Education level				
Not educated	3 (1.6)	–	3 (1.9)	0.670
Primary school	17 (9.2)	3 (11.1)	14 (8.9)	
Secondary school	7 (3.8)	2 (7.4)	5 (3.2)	
High school	50 (27.0)	6 (22.2	44 (27.8)	
Graduate	68 (36.8)	12 (44.4	56 (35.4)	
Post graduate	40 (21.6)	4 (14.8	36 (22.8)	
Residence				
Rural	56 (30.3)	8 (29.6)	48 (30.4)	0.938
Urban	129 (69.7)	19 (70.4)	110 (69.6)	
Employment status				
Not employed	65 (35.1)	6 (22.2	59 (37.3)	0.468
Employed	97 (52.4)	17 (63.0)	80 (50.6)	
Retired	4 (2.2)	1 (3.7)	3 (1.9)	
Student	19 (10.3)	3 (11.1)	16 (10.1)	
Socioeconomic status				
Low	45 (24.3)	5 (18.5)	40 (25.3)	0.649
Moderate	136 (73.5)	21 (77.8)	115 (72.8)	
High	4 (2.2)	1 (3.7)	3 (1.9)	
Life habits				
Smoking	39 (21.1)	6 (22.2)	33 (20.9)	0.875
Alcohol consumption	1 (0.5)	–	1 (0.6)	1.000
Exercise practice	51 (27.6)	9 (33.3)	42 (26.6)	0.468
Treatment payment system				
Health insurance	60 (32.4)	6 (22.2)	54 (34.2)	0.220
State expense	49 (26.5)	7 (25.9)	42 (26.6)	0.943
Patient expense	150 (81.1)	19 (70.4)	131 (82.9	0.124

Bold values indicate statistical significant p values (p < 0.05)

The mean age (SD) at BD onset was 28.35 (9.77) years, with a median duration of 4 months of diagnosis delay. The most prevalent clinical manifestation was recurrent oral aphthosis (95.1%), followed by ocular involvement (72.4%) and recurrent genital aphthosis (67%). Regarding administered drugs, colchicine (87.0%) was the most used, followed by corticosteroids (77.3%) and azathioprine (47.6%). The age at disease onset (p = 0.007) showed a statistically significant difference between those with MSK manifestations and those without. Almost always, all cumulative clinical manifestations of BD were more prevalent in those with MSK manifestations than those without, as shown in Table 2.

**Table 2:** Clinical and therapeutic data of the study BD patients (n = 185)

Variable n (%), median (min–max), mean ± SD	Total BD patients (n = 185)	Musculoskeletal manifestations		p
		Without (n = 27) 14.6%	With (n = 158) 85.4%	
Clinical data of Behcet disease				
Age at disease onset (years)	28.35 ± 9.77	24.62 ± 6.80	29.00 ± 10.07	**0.007**
Diagnostic delay (months)	12 (0–96)	10 (1–90)	12 (0–96)	0.100
Disease duration (years)	4 (1–29)	3 (1–20)	4 (1–29)	0.898
Family history of Behcet disease	29 (15.7)	4 (14.8)	25 (15.8)	0.894
Cumulative manifestations of Behcet disease				
Recurrent oral aphthosis	176 (95.1)	26 (96.3)	150 (94.9)	1.000
Erythema nodosum	79 (42.7)	3 (11.1)	76 (48.1)	** < 0.001**
Pseudofolliculitis	84 (45.4)	6 (22.2)	78 (49.4)	**0.009**
Skin ulcers	105 (56.8)	8 (29.6)	97 (61.4)	**0.002**
Recurrent genital aphthosis	124 (67.0)	15 (55.6)	109 (69.0)	0.170
Recurrent attacks of fever	121 (65.4	13 (48.1)	108 (68.4)	**0.041**
Abdominal pain/nausea/diarrhoea	123 (66.5	9 (33.3)	114 (72.2)	** < 0.001**
Ocular involvement	134 (72.4)	11 (40.7)	123 (77.8)	** < 0.001**
CNS manifestations	35 (18.9)	2 (7.4)	33 (20.9)	0.116
Tingling and numbness	86 (46.5)	4 (14.8)	82 (51.9)	** < 0.001**
Vascular involvement	109 (58.9)	8 (29.6)	101 (63.9)	**0.001**
Cardiac involvement	57 (30.8)	1 (3.7)	56 (35.4)	** < 0.001**
Arrhythmia	82 (44.3)	2 (7.4)	80 (50.6)	** < 0.001**
Lymphadenopathy	37 (20.0)	3 (11.1)	34 (21.5)	0.299
Pleuritis	22 (11.9)	–	22 (13.9)	**0.048**
Orchitis	26 (14.1)	4 (14.8)	22 (13.9)	1.000
BD therapeutic data				
Colchicine	161 (87.0)	22 (81.5)	139 (88.0)	0.353
Corticosteroids	143 (77.3)	17 (63.0)	126 (79.7)	**0.054**
Antimalarials	20 (10.8)	1 (3.7)	19 (12.0)	0.317
Azathioprine	88 (47.6)	10 (37.0)	78 (49.4)	0.236
Sulfasalazine	11 (5.9)	1 (3.7)	10 (6.3)	1
Methotrexate	19 (10.3)	3 (11.1)	16 (10.1)	0.876
Mycophenolate	13 (7.0)	1 (3.7)	12 (7.6)	0.696
Cyclosporine	14 (7.6)	–	14 (8.9)	0.228
Biologics				
Adalimumab	23 (12.4)	2 (7.4)	21 (13.3)	0.537
Etanercept	3 (1.6)	–	3 (1.9)	1
Infliximab	12 (6.5)	1 (3.7)	11 (7.0)	1
Rituximab	9 (4.9)	–	9 (5.7)	0.361

Bold values indicate statistical significant p values (p < 0.05)

**Fig. 2:**
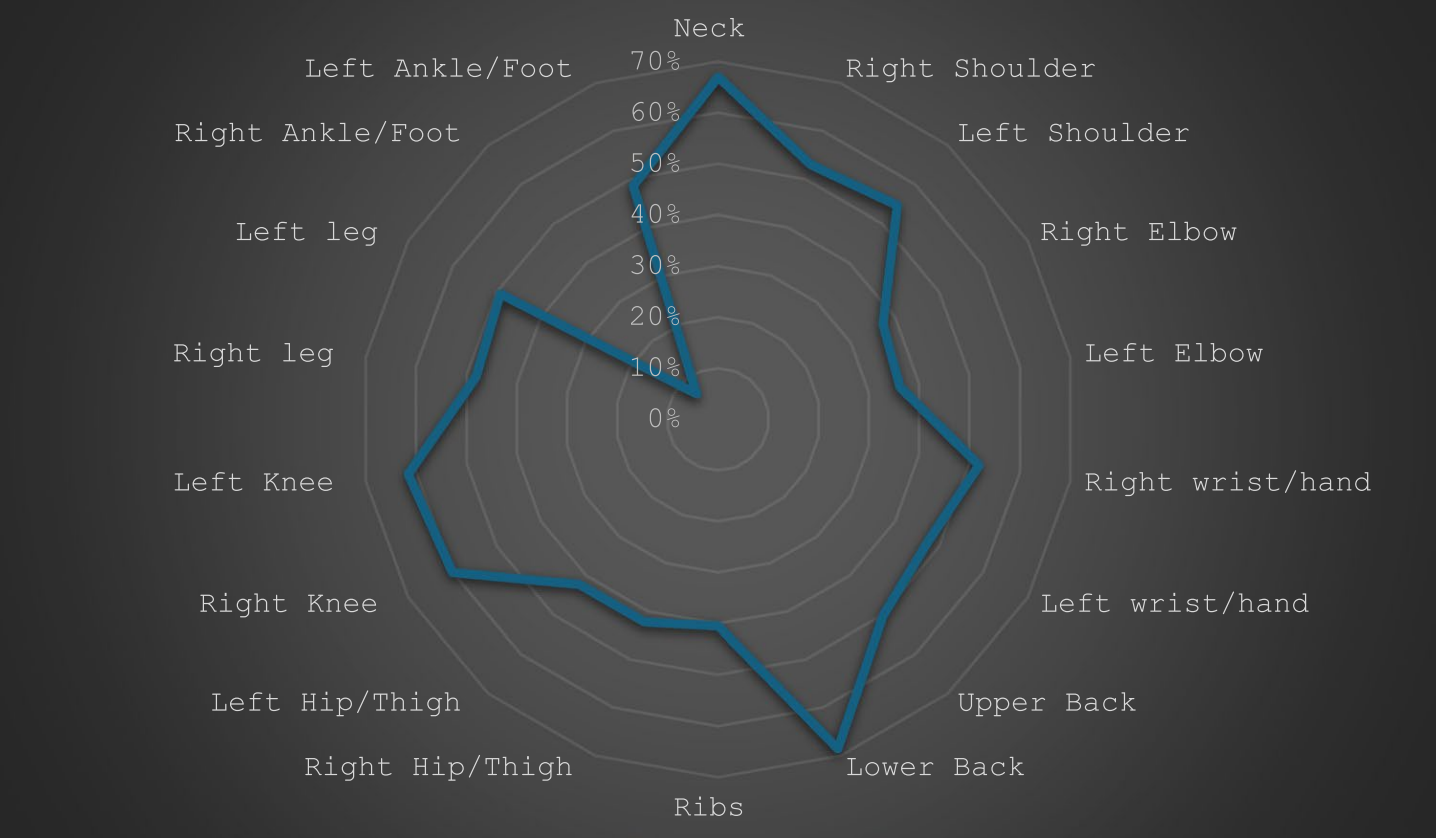
MSK symptoms in the last 6 months duration using Nordic MSK Questionnaire among the study BD patients (*n* = 185)

Figure 2 displays the frequency and distribution of MSK symptoms (pain, numbness, discomfort, and numbness) throughout the preceding 6 months. Most patients reported pain in the lower back (69%) and neck (67%), followed by the left and right knees (62% and 60%, respectively), while the least affected areas of the body were the right elbow (37%) and the right ankle and foot (7%).

According to the Nordic MSK questionnaire, most of the studied patients (85.4%) reported MSK symptoms in the last 6 months, with 1.6% describing at least one site of discomfort, 0.5% describing at least two, 3.8% describing at least three, 4.9% describing at least four, and 74.6% describing more than four body sites of MSK discomfort as illustrated in Table 3. For 41.1% of patients, these MSK manifestations persisted for over 3 months.

**Table 3:** Number and duration of MSK discomforts per individual in the last 6 months using Nordic MSK Questionnaire among the study BD patients (*n* = 185)

Variable	Studied group (*n* = 185), *n* (%)
MSK discomfort	
No discomfort	27 (14.6)
At least 1 discomfort	3 (1.6)
At least 2 discomforts	1 (0.5)
At least 3 discomforts	7 (3.8)
At least 4 discomforts	9 (4.9)
> 4 discomforts	138 (74.6)
Duration MSK discomfort	
< 6 weeks	64 (34.6)
6 weeks–3 months	18 (9.7)
≥ 3 months	76 (41.1)

Table 4 summarizes the impact of MSK symptoms on HRQoL in BD patients, as assessed by the SF-36 questionnaire. The findings indicate that BD patients with MSK symptoms experience significantly lower scores across all SF-36 domains compared to those without, reflecting greater physical, emotional, and social impairments. Notably, PF, role RP, and RE were severely affected (p < 0.001), highlighting substantial limitations in daily activities. Additionally, VT and MH scores were significantly lower in the MSK group (p < 0.001 and p = 0.004, respectively). SF and BP scores also showed a marked decline in affected patients (p < 0.001). Lastly, GH scores were significantly lower in BD patients with MSK symptoms (p = 0.018).

**Table 4:** Distribution of SF36 domains according to the presence or absence of MSK symptoms in the last 6 months according to Nordic Questionnaire (*n* = 185)

Variables median (min–max)	Total BD patients (n = 185)	Musculoskeletal manifestations		p
		Without (n = 27)	With (n = 158)	
Physical functioning (PF)	60 (0–100)	90 (0–100)	55 (0–100)	** < 0.001**
Role functioning (due to physical condition) (RP)	0 (0–100)	100 (0–100)	0 (0–100)	** < 0.001**
Role functioning/emotional (RE)	0 (0–100)	100 (0–100)	0 (0–100)	** < 0.001**
Energy/fatigue (VT)	40 (0–90)	50 (15–90)	40 (0–70)	** < 0.001**
Emotional well-being(MH)	40 (0–96)	52 (8–84)	40 (0–96)	**0.004**
Social functioning (SF)	50 (0–100)	87.5 (25–100)	50 (0–100)	** < 0.001**
Pain (BP)	45 (0–100)	100 (0–100)	35 (0–100)	** < 0.001**
General health (GH)	40 (10–85)	45 (20–85)	40 (10–75)	**0.018**

Bold values indicate statistical significant p values (p < 0.05)

## Discussion

To the best of our knowledge, this is the first comprehensive study conducted on BD patients that used a Nordic MSK questionnaire to assess MSK manifestations. The study involves BD patients and collects data on MSK manifestations and their impact on HRQoL in these patients. The study found that BD patients experienced high MSK symptom load, leading to lower HRQoL scores in all domains.

BD is typically recognized as a disorder with a male predominance; however, the male-to-female ratio may differ across endemic and non-endemic regions [23]. BD in Silk Route regions, exhibiting a male-to-female ratio of approximately 2–10:1; in contrast, the ratio approaches 1:1 in Western Europe and the United States [24]. In the present work, the ratio between female and male is 1.2:1. Male-to-female ratios range from 0.63 in Korea to 0.98 in Japan, 1.3 in Iran, and 1.34 in China [25]. The 2020 cohort study conducted in the United Kingdom observed a higher prevalence in females (63.8%) [26].

Even though BD can manifest at any age, most reports indicate that the mean age of onset is in the third decade of life. Typically, BD commences with a single manifestation. Several months later, the second manifestation occurs, and so forth [23]. The mean age at BD onset in our cohort was 28.35 years. The mean age of disease onset was recorded at 26.7 years for men and 28.4 years for women in the German registry of BD [27]. The onset occurring before the age of 15 or after the age of 50 to 55 years is considered to be relatively uncommon [28]. In the nationwide epidemiological study of BD in Korea, fewer than 3% of all identified incident cases were elderly patients (aged 70 years or older) [29]. The percentage of juvenile onset cases in various cohorts of BD was estimated to be between 2 and 5% [30].

According to the Nordic MSK questionnaire, most of the patients studied (85.4%) reported MSK manifestations. In fact, arthropathy is frequent in BD, with a prevalence rate ranging from 11 to 93% [31, 32]. Articular involvement was observed in 37.4% of Iranian patients with BD [33]. Arthritis is observed at the time of diagnosis in approximately 70% of patients, while 9% of patients exhibit arthritis only as the primary manifestation [34]. Arthritis in BD is often non-erosive. Other infrequent MSK manifestations of BD include destructive arthritis, pseudo-gout, and fibromyalgia [35].

Most of our patients reported pain in the lower back (69%). Sacroiliitis may be the cause of this low back pain. The literature contains conflicting findings regarding the prevalence of sacroiliac joint (SIJ) involvement in BD. Dilsen et al. [36] reported the maximum values of SIJ involvement in BD at first in 1979 (63%) and then in 1986 (43%). According to Caporn et al. [32], five of fourteen patients with BD in Britain were diagnosed with erosive sacroiliitis in 1983. In a study of 20 BD patients, Olivieri et al. [37] recorded a 30% prevalence of SIJ involvement (four patients). There is also ongoing dispute about whether BD is one of the seronegative spondyloarthropathies (SpA) or if the SIJ involvement is a hallmark of BD [38]. Some publications have observed a high prevalence of SIJ involvement in BD, whereas others have shown an even prevalence with healthy controls [39].Although joint involvement of BD and spondyloarthropathies is comparable, sacroiliitis and HLA-B*27 positive are rare [40].

Joint involvement in BD, resulting in recurrent migratory arthritis, synovitis, and joint degeneration, predominantly affecting the knee [41] and commonly characterized by asymmetric polyarticular arthralgias of the knees, hips, and elbows [42]. Also,an increase in neck pain complaints among BD patients was noted [43]. Yafei Cui et al. reported a case of a patient with BD whose main complaint was recurring neck pain resulting from atlanto-axial dislocation [44].In the present study, neck and knee pain were among the most reported regions of discomfort among BD patients.

Most of the patients studied reported more than four body areas of MSK manifestations. Polyarthritis typically affects the larger limb joints as well as small joints of the hands and feet [13]. It was reported that about 5% of the patients had polyarticular involvement [45]. Examination of synovial fluid during acute episodes demonstrates inflammatory fluid characterized by elevated viscosity and leukocyte counts (ranging from 5000 to 50,000, with a predominance of polymorphonuclear leukocytes), alongside normal protein and glucose levels [46].

Gender variations in BS have been the subject of numerous studies. There were notable gender-specific differences found in a research encompassing BS patients from various populations [47]. MSK manifestations were significantly higher among females in our cohort. This is consistent with findings in the general population. The fact that females complain more than males do and frequently report more pertinent health information due to social and educational issues as well as the presence of hormone changes provides an answer to the gender question [48].

Cumulative manifestations are significantly higher among those with MSK manifestations. Previous research has linked arthritis to papulopustular lesions, which are frequently infected with microorganisms such as staphylococci and Prevotella, and an elevated risk of enthesopathy in these patients [40].

The HRQoL in patients with BD is adversely impacted by the disease [49].In the present work, patients exhibiting MSK manifestations demonstrated statistically significant lower scores across all domains compared to those without such manifestations. As BD is a chronic condition characterized by a relapsing course, the related complaints negatively impact both physical and mental health, hence diminishing HRQoL [50]. Canpolat et al. [16] also reported that patients with BD had low HRQoL scores, which were influenced by complaints of arthritis and body pain.

In the present study, there are a few limitations that need to be addressed. Because the data were acquired primarily by self-reporting, it is possible that the findings of the study were influenced by the method that was used to collect them. One important limitation of this study is the absence of a control group comprising age- and sex-matched healthy individuals and/or patients with other chronic diseases such as glaucoma or diabetic retinopathy. The inclusion of a control group would have provided a comparative perspective on the impact of MSK manifestations and HRQoL in BD patients relative to other conditions. Also, NMQ-E does not differentiate between inflammatory and non-inflammatory pain, which is particularly relevant in BD. Although BD diagnosis was confirmed by an expert rheumatologist, no clinical assessments were performed to rule out other potential causes of MSK pain, such as fibromyalgia or osteoarthritis, which are common in the general population. As a result, it is not possible to definitively attribute MSK symptoms solely to BD, and this should be interpreted with caution. Another limitation of this study is the lack of physical measures, such as body mass index, which can impact MSK symptoms and HRQoL in BD. Obesity may exacerbate MSK pain through increased joint stress, potentially influencing results. Additionally, mood disturbances, including anxiety and depression, were not assessed using standardized tools, despite their known role in pain perception.

In conclusion, the findings of this study show a significant prevalence of MSK symptoms among BD patients, as well as a negative impact on HRQoL. The lower back and neck appear to be the most commonly affected areas of the body. Furthermore, longitudinal multicenter studies are needed to examine, detect, and treat MSK symptoms in BD patients.

## Data Availability

The datasets used and/or analysed during the current study are available from the corresponding author on reasonable request.

## Abbreviations

BD: Behçet’s disease
bDMARDs: Biological disease-modifying antirheumatic drugs
BP: Bodily pain
cDMARDs: Conventional disease-modifying antirheumatic drugs
GH: General health
HRQoL: Health-related quality of life
MH: Mental health
MSK: Musculoskeletal
NMQ-E: Nordic MSK questionnaire
PF: Physical functioning
RE: Role limitation due to emotional problems
RP: Role limitation due to physical functioning
SF: Social functioning
SF-36: SF-36
SIJ: Sacroiliac joint
VT: Vitality

## References

[1] Sakane T, Takeno M, Suzuki N, Inaba G (1999) Behçet’s disease. N Engl J Med 341:1284–1291. 10.1056/NEJM199910213411707 10528040 10.1056/NEJM199910213411707

[2] Yurdakul S (2020) Epidemiology of Behçet syndrome and regional differences in disease expression. In: Yazici Y, Hatemi G, Seyahi E, Yazici H (eds) Behçet syndrome. Springer International Publishing, Cham, pp 21–35

[3] Hirohata T, Kuratsune M, Nomura A, Jimi S (1975) Prevalence of Behçet’s syndrome in Hawaii. With particular reference to the comparison of the Japanese in Hawaii and Japan. Hawaii Med J 34:244–246 1165185

[4] Madanat WY, Alawneh KM, Smadi MM et al (2017) The prevalence of Behçet’s disease in the north of Jordan: a hospital-based epidemiological survey. Clin Exp Rheumatol 35(Suppl 108):51–54 29224587

[5] Yazici H, Fresko I, Yurdakul S (2007) Behçet’s syndrome: disease manifestations, management, and advances in treatment. Nat Clin Pract Rheumatol 3:148–155. 10.1038/ncprheum0436 17334337 10.1038/ncprheum0436

[6] Li C, Li L, Wu X et al (2020) Clinical manifestations of Behçet’s disease in a large cohort of Chinese patients: gender- and age-related differences. Clin Rheumatol 39:3449–3454. 10.1007/s10067-020-05026-2 32394216 10.1007/s10067-020-05026-2

[7] Mendes D, Correia M, Barbedo M et al (2009) Behçet’s disease—a contemporary review. J Autoimmun 32:178–188. 10.1016/j.jaut.2009.02.011 19324519 10.1016/j.jaut.2009.02.011

[8] Fatemi A, Shahram F, Akhlaghi M et al (2017) Prospective study of articular manifestations in Behçet’s disease: five-year report. Int J Rheum Dis 20:97–102. 10.1111/1756-185X.12633 26111117 10.1111/1756-185X.12633

[9] Kötter I, Lötscher F (2021) Behçet’s syndrome apart from the triple symptom complex: vascular, neurologic, gastrointestinal, and musculoskeletal manifestations. A mini review. Front Med 8:639758. 10.3389/fmed.2021.639758 10.3389/fmed.2021.639758 PMC8063110 33898481

[10] Dawson AP, Steele EJ, Hodges PW, Stewart S (2009) Development and test-retest reliability of an extended version of the Nordic Musculoskeletal Questionnaire (NMQ-E): a screening instrument for musculoskeletal pain. J Pain 10:517–526. 10.1016/j.jpain.2008.11.008 19345154 10.1016/j.jpain.2008.11.008

[11] Tharwat S, Husain SM (2024) Musculoskeletal symptoms in systemic lupus erythematosus patients and their impact on health-related quality of life. BMC Musculoskelet Disord 25:272. 10.1186/s12891-024-07367-4 38589834 10.1186/s12891-024-07367-4 PMC11003043

[12] Tharwat S, Nassar MK (2024) Musculoskeletal symptoms and their impact on health-related quality of life in chronic nonbacterial osteomyelitis patients. Pediatr Rheumatol Online J 22:34. 10.1186/s12969-024-00971-7 38448884 10.1186/s12969-024-00971-7 PMC10916259

[13] Bicer A (2011) Musculoskeletal findings in Behcet’s disease. Pathol Res Int 2012:653806. 10.1155/2012/653806 10.1155/2012/653806 PMC3180072 21961082

[14] Pugh JD, Gelder L, Williams AM et al (2015) Validity and reliability of an online extended version of the Nordic Musculoskeletal Questionnaire (NMQ-E2) to measure nurses’ fitness. J Clin Nurs 24:3550–3563. 10.1111/jocn.12971 26415886 10.1111/jocn.12971

[15] Pinquart M (2020) Health-related quality of life of young people with and without chronic conditions. J Pediatr Psychol 45:780–792. 10.1093/jpepsy/jsaa052 32642762 10.1093/jpepsy/jsaa052

[16] Canpolat O, Yurtsever S (2011) The quality of life in patients with Behçet’s disease. Asian Nurs Res 5:229–235. 10.1016/j.anr.2011.12.003 10.1016/j.anr.2011.12.003 25030525

[17] Onal S, Savar F, Akman M, Kazokoglu H (2010) Vision- and health-related quality of life in patients with Behçet uveitis. Arch Ophthalmol Chic Ill 1960 128:1265–1271. 10.1001/archophthalmol.2010.209 10.1001/archophthalmol.2010.209 20937995

[18] Mastrolia MV, Marinello D, di Cianni F, et al (2022). Assessing quality of life in Behçet’s disease: a systematic review. Clin Exp Rheumatol. 40:1560–1566. 10.55563/clinexprheumatol/sian1b 10.55563/clinexprheumatol/sian1b 36106544

[19] International Team for the Revision of the International Criteria for Behçet’s Disease (ITR-ICBD) (2014) The International Criteria for Behçet’s Disease (ICBD): a collaborative study of 27 countries on the sensitivity and specificity of the new criteria. J Eur Acad Dermatol Venereol JEADV 28:338–347. 10.1111/jdv.12107 23441863 10.1111/jdv.12107

[20] Kuorinka I, Jonsson B, Kilbom A et al (1987) Standardised Nordic questionnaires for the analysis of musculoskeletal symptoms. Appl Ergon 18:233–237. 10.1016/0003-6870(87)90010-x 15676628 10.1016/0003-6870(87)90010-x

[21] Aldhabi R, Albadi M, Kahraman T, Alsobhi M (2024) Cross-cultural adaptation, validation and psychometric properties of the Arabic version of the Nordic Musculoskeletal Questionnaire in office working population from Saudi Arabia. Musculoskelet Sci Pract 72:103102. 10.1016/j.msksp.2024.103102 38896911 10.1016/j.msksp.2024.103102

[22] AboAbat A, Qannam H, Bjorner JB, Al-Tannir M (2020) Psychometric validation of a Saudi Arabian version of the sf-36v2 health survey and norm data for Saudi Arabia. J Patient-Rep Outcomes 4:67. 10.1186/s41687-020-00233-6 32789705 10.1186/s41687-020-00233-6 PMC7426352

[23] Davatchi F, Shahram F, Chams-Davatchi C et al (2010) Behcet’s disease: from East to West. Clin Rheumatol 29:823–833. 10.1007/s10067-010-1430-6 20354748 10.1007/s10067-010-1430-6

[24] Lin Y-H, Tai T-Y, Pu C-Y et al (2018) Epidemiology of Behcet’s disease in Taiwan: a population-based study. Ophthalmic Epidemiol 25:323–329. 10.1080/09286586.2018.1469157 29726724 10.1080/09286586.2018.1469157

[25] Cansu DÜ, Kaşifoğlu T, Korkmaz C (2016) Do clinical findings of Behçet’s disease vary by gender?: a single-center experience from 329 patients. Eur J Rheumatol 3:157–160. 10.5152/eurjrheum.2016.038 28149658 10.5152/eurjrheum.2016.038 PMC5283562

[26] Thomas T, Chandan JS, Subramanian A et al (2020) Epidemiology, morbidity and mortality in Behçet’s disease: a cohort study using the Health Improvement Network (THIN). Rheumatol Oxf Engl 59:2785–2795. 10.1093/rheumatology/keaa010 10.1093/rheumatology/keaa010 32040196

[27] Bonitsis NG, Luong Nguyen LB, LaValley MP et al (2015) Gender-specific differences in Adamantiades-Behçet’s disease manifestations: an analysis of the German registry and meta-analysis of data from the literature. Rheumatol Oxf Engl 54:121–133. 10.1093/rheumatology/keu247 10.1093/rheumatology/keu247 25118314

[28] Mahr A, Maldini C (2014) Epidemiology of Behçet’s disease. Rev Med Interne 35:81–89. 10.1016/j.revmed.2013.12.005 24398415 10.1016/j.revmed.2013.12.005

[29] Lee YB, Lee SY, Choi JY et al (2018) Incidence, prevalence, and mortality of Adamantiades-Behçet’s disease in Korea: a nationwide, population-based study (2006–2015). J Eur Acad Dermatol Venereol JEADV 32:999–1003. 10.1111/jdv.14601 28940547 10.1111/jdv.14601

[30] Vaiopoulos AG, Kanakis MA, Kapsimali V et al (2016) Juvenile Adamantiades-Behçet disease. Dermatol Basel Switz 232:129–136. 10.1159/000442667 10.1159/000442667 26736030

[31] Salvarani C, Pipitone N, Catanoso MG et al (2007) Epidemiology and clinical course of Behçet’s disease in the Reggio Emilia area of Northern Italy: a seventeen-year population-based study. Arthritis Rheum 57:171–178. 10.1002/art.22500 17266063 10.1002/art.22500

[32] Caporn N, Higgs ER, Dieppe PA, Watt I (1983) Arthritis in Behcet’s syndrome. Br J Radiol 56:87–91. 10.1259/0007-1285-56-662-87 6824840 10.1259/0007-1285-56-662-87

[33] Davatchi F, Shahram F, Chams-Davatchi C et al (2010) Behcet’s disease in Iran: analysis of 6500 cases. Int J Rheum Dis 13:367–373. 10.1111/j.1756-185X.2010.01549.x 21199472 10.1111/j.1756-185X.2010.01549.x

[34] Chamberlain MA (1977) Behcet’s syndrome in 32 patients in Yorkshire. Ann Rheum Dis 36:491–499. 10.1136/ard.36.6.491 596943 10.1136/ard.36.6.491 PMC1000153

[35] Yavuz S, Fresko I, Hamuryudan V et al (1998) Fibromyalgia in Behçet’s syndrome. J Rheumatol 25:2219–2220 9818667

[36] Dilsen N, Konice M, Ovul C (1979) Arthritic patterns in Behçet’s disease. Behçs Dis Amst Oxf Excerpta Medica 145–55

[37] Olivieri I, Gemignani G, Camerini E et al (1990) Computed tomography of the sacroiliac joints in four patients with Behçet’s syndrome–confirmation of sacroiliitis. Br J Rheumatol 29:264–267. 10.1093/rheumatology/29.4.264 2379043 10.1093/rheumatology/29.4.264

[38] Chang HK, Lee DH, Jung SM et al (2002) The comparison between Behçet’s disease and spondyloarthritides: does Behçet’s disease belong to the spondyloarthropathy complex? J Korean Med Sci 17:524–529. 10.3346/jkms.2002.17.4.524 12172050 10.3346/jkms.2002.17.4.524 PMC3054915

[39] Kotevoglu N, Tasbas I, Bekiroglu N (2004) Computed tomography does not support sacroiliitis as a feature of behçet disease: a metaanalytic review. J Clin Rheumatol Pract Rep Rheum Musculoskelet Dis 10:42–45. 10.1097/01.rhu.0000111298.67743.08 10.1097/01.rhu.0000111298.67743.08 17043460

[40] Hatemi G, Bahar H, Uysal S et al (2004) The pustular skin lesions in Behcet’s syndrome are not sterile. Ann Rheum Dis 63:1450–1452. 10.1136/ard.2003.017467 15479894 10.1136/ard.2003.017467 PMC1754819

[41] Yazici Y, Hatemi G, Bodaghi B et al (2021) Behçet syndrome. Nat Rev Dis Primer 7:67. 10.1038/s41572-021-00301-1 10.1038/s41572-021-00301-1 34531393

[42] Mazzoccoli G, Matarangolo A, Rubino R et al (2016) Behçet syndrome: from pathogenesis to novel therapies. Clin Exp Med 16:1–12. 10.1007/s10238-014-0328-z 25447032 10.1007/s10238-014-0328-z

[43] Manav V, İlhan D, Mercan H et al (2022) Association between intervertebral disc degeneration and Behçet’s disease. Dermatol Ther 35:e15585. 10.1111/dth.15585 35569115 10.1111/dth.15585

[44] Komatsumoto M, Kono M, Shimizu Y (2021) A case of Behçet’s syndrome initially presenting as recurrent neck pain. Rheumatol Oxf Engl 60:e12–e13. 10.1093/rheumatology/keaa376 10.1093/rheumatology/keaa376 32780834

[45] Hatemi G, Fresko I, Yurdakul S et al (2010) Reply to letter by Priori et al commenting on whether Behçet’s syndrome patients with acne and arthritis comprise a true subset. Arthritis Rheum 62:305–306. 10.1002/art.27181 20039415 10.1002/art.27181

[46] Ceccarelli F, Priori R, Iagnocco A et al (2007) Knee joint synovitis in Behçet’s disease: a sonographic study. Clin Exp Rheumatol 25:S76-79 17949556

[47] Jo YG, Ortiz-Fernández L, Coit P et al (2022) Sex-specific analysis in Behçet’s disease reveals higher genetic risk in male patients. J Autoimmun 132:102882. 10.1016/j.jaut.2022.102882 35987173 10.1016/j.jaut.2022.102882 PMC10614427

[48] Yao W, Luo C, Ai F, Chen Q (2012) Risk factors for nonspecific low-back pain in Chinese adolescents: a case-control study. Pain Med Malden Mass 13:658–664. 10.1111/j.1526-4637.2012.01369.x 10.1111/j.1526-4637.2012.01369.x 22494366

[49] Khabbazi A, Ebrahimzadeh Attari V, Asghari Jafarabadi M, Malek Mahdavi A (2021) Quality of life in patients with Behçet disease and its relation with clinical symptoms and disease activity. Reumatol Clin 17:1–6. 10.1016/j.reuma.2019.03.002 31078452 10.1016/j.reuma.2019.03.002

[50] Bernabé E, Marcenes W, Mather J et al (2010) Impact of Behçet’s syndrome on health-related quality of life: influence of the type and number of symptoms. Rheumatol Oxf Engl 49:2165–2171. 10.1093/rheumatology/keq251 10.1093/rheumatology/keq251 20675710

